# KeyCap3D: Keyword-Guided 3D Medical Image Captioning with Cross-Attention

**DOI:** 10.1016/j.mex.2026.103890

**Published:** 2026-03-28

**Authors:** Supriyanto Supriyanto, Muhammad Ibadurrahman Arrasyid Supriyanto, Haviluddin Haviluddin, Chalsi Mala Sari, Hajar Mar'atussholikah Supriyanto, Rayner Alfred

**Affiliations:** a Faculty of Mathematics and Natural Science, Mulawarman University, Indonesia; b Faculty of Engineering, Mulawarman University, Indonesia; c Faculty of Medicine, Muhammadiyah University of East Kalimantan, Indonesia; d Faculty of Computing and Informatics, Universiti Malaysia Sabah, Malaysia

**Keywords:** Automated captioning, Keyword-guided model, Brain tumor detection, Glioblastoma, 3D FLAIR MRI

## Abstract

This study presents a keyword-guided cross-attention framework for automated radiological report generation from 3D FLAIR MRI brain tumor images. The architecture integrates M3D-CLIP as the image encoder. Hierarchical keyword extraction is performed using fine-tuned KeyBERT and BioBERT semantic embeddings in a 768-dimensional space. Six cross-attention layers fuse visual features with clinical keywords across four hierarchical levels: abnormality type, lesion characteristics, anatomical location, and lateralization. A four-layer transformer decoder generates captions autoregressively. The BraTS2020 dataset containing 369 glioma patients paired with TextBraTS radiological descriptions was preprocessed with center-focused slice selection of 32 from 155 slices and spatial interpolation to 256 × 256 resolution. Training on NVIDIA RTX 3050 GPU for 15 epochs using AdamW optimizer achieved loss reduction from 4.16 to 1.33. Evaluation on 20 test samples demonstrated BLEU-1 of 0.5359, BLEU-2 of 0.3969, and ROUGE-L of 0.5051, with generated captions accurately capturing clinical information for decision support applications.

• Multi-modal fusion through keyword-guided cross-attention integrating visual MRI features with hierarchical clinical terminology
• Transformer-based autoregressive generation conditioned on enriched image-keyword representations
• Comprehensive evaluation using BLEU and ROUGE metrics on brain tumor caption generation task

## Graphical abstract

**Figure:**
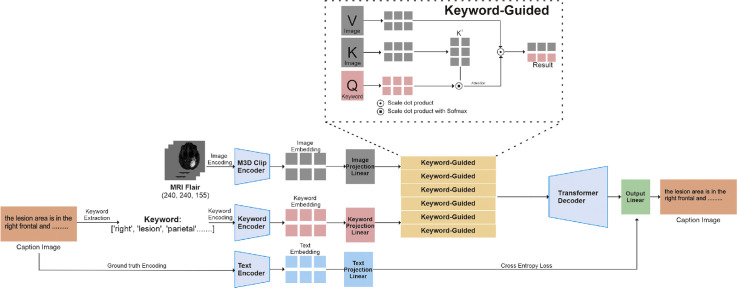
Proposesd Keyword-Guided 3D Medical Image Captioning Architecture.

**Table:** Specifications table.

**Subject area**	Neuroscience
**More specific subject area**	Medical Text to Text Generation, Vision-Language Models
**Name of your method**	KeyCap3D
**Name and reference of original method**	A. Vaswani et al., “Attention is All you Need,” in *Advances in Neural Information Processing Systems*, Curran Associates, Inc., 2017.
**Resource availability**	Hardware: NVIDIA GPU with ≥16GB VRAM Software: PyTorch 2.0+, Transformers 4.30+, Python 3.10+

## Background

Brain and central nervous system cancer represents a significant global public health burden due to its high mortality and low survival rates. Based on data from the Global Burden of Disease study, a total of 347,992 new cases of brain cancer and 246,253 deaths were recorded worldwide in 2019, indicating a substantial mortality burden associated with this disease [1]. Despite advances in diagnostic and therapeutic strategies, survival outcomes for brain cancer remain poor. The diagnosis of brain cancer relies heavily on advanced imaging techniques, which are more accessible in high-income regions and contribute to regional differences in incidence and mortality rates. Accurate diagnosis and assessment are essential for effective treatment planning, as delayed diagnosis and limited access to appropriate imaging and treatment are associated with poorer outcomes.

To overcome the high burden of medical image interpretation, medical image captioning has been developed as a technology that generates automatic text descriptions of medical images using artificial intelligence [2]. Unlike classification systems that only determine the presence or absence of tumors or segmentation systems that mark tumor boundaries, captioning technology generates complete sentences that describe the location of the tumor, its characteristic shape, and its effects on surrounding tissue [3]. Advances in deep learning have enabled the adaptation of architectures convolutional neural networks to process images and long short-term memory to generate texton a chest X-ray dataset [4]. However, the application of medical image captioning for brain tumors faces three main challenges. First, brain MRI data is volumetrically complex with multi-parametric sequences across millions of voxels [5] . Second, describing the location of a tumor requires the identification of multiple brain structures in a hierarchical manner, from the hemispheres and lobes to individual gyri [6], following established anatomical atlases . Third, brain tumors, particularly glioblastomas, exhibit significant morphological heterogeneity with varying shapes from well-circumscribed to infiltrative margins with ill-defined boundaries making automated recognition challenging for computational models [7].

Several studies have developed medical image captioning approaches to address the challenges of brain tumor image interpretation. In study [4], researchers proposed a method of compressing images into a single global vector using a CNN encoder and LSTM decoder with an attention mechanism that achieved a BLEU-4 score of 0.28 on chest X-rays. The main drawback is that the attention mechanism lacks anatomical guidance, so the model cannot accurately determine anatomical locations. In study [8], researchers addressed the problem of location information loss using ResNet-101 and a memory-driven transformer, achieving a BLEU-4 score of 0.353 on chest X-ray images. The main drawback is that compressing 8.9 million image points into 768 numbers removes anatomical position coordinate information, preventing the model from accurately distinguishing anatomical locations. In study [9], researchers improved the global encoding problem by using hierarchical classification for semantic feature extraction and then integrating it with Vision Transformer and BioMedBERT, achieving a BLEU-1 score of 0.3348 on MRI brain images. The main drawback is the use of the BLEU-1 evaluation metric, which only measures unigram precision and cannot evaluate word sequence accuracy, so that medical phrases such as “right frontal lobe” and “frontal right lobe” receive the same score even though they have different anatomical meanings.

To overcome the problem of spatial information loss, this study proposes KeyCap3d (Keyword-Guided 3D Medical Image Captioning with Cross-Attention) through three integrated innovations. First, M3D-CLIP extracts visual features of each tumor region independently. Second, keyword extraction uses KeyBert to encode medical terms in four levels, namely the type of abnormality, lesion characteristics, anatomical location, and lateralization. Third, keyword-guided cross-attention uses keywords as a guide in the attention mechanism to give high weight to relevant visual regions so that the resulting textual description corresponds to the correct anatomical location. Validation was performed on the TextBraTS dataset using the BLEU-4 and ROUGE-L evaluation metrics.

## Method details

**Fig. 1:**
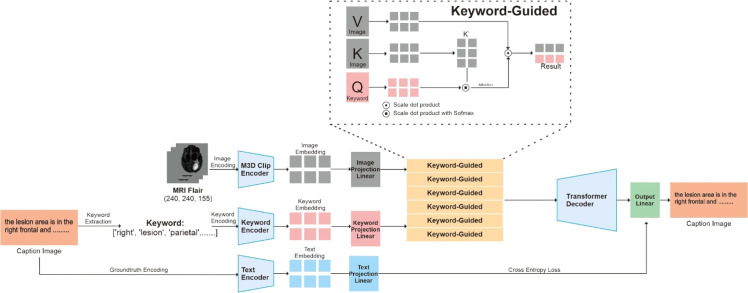
KeyCap3D model.

We propose a cross-attention-based medical image captioning framework that generates natural language descriptions of brain tumors from 3D MRI FLAIR sequences. The method consists of: (1) feature extraction using M3D Clip encoder, (2) multi-modal fusion through cross-attention layers between image features and keyword embeddings, and (3) caption generation via transformer decoder. The model is trained end-to-end with cross-entropy loss to produce clinically accurate descriptions. This architecture diagram shows in Fig. 1:

### Dataset and data preprocessing

In this study, we used two datasets. First, 3D FLAIR MRI volumes were obtained from the BraTS2020 (Brain Tumor Segmentation) dataset, which consists of brain MRI sequences from 369 glioma patients with original dimensions of 240 × 240 × 155 voxels [10]. Second, the caption data used was sourced from TextBraTS, a specialized dataset that provides radiological descriptions for brain tumor MRI in the BraTS2020 dataset [11].

To adjust the 3D MRI data to the input requirements of the M3D Clip encoder (32 × 256 × 256), we applied a strategic slice selection and pre-processing pipeline. First, we applied min-max normalization to standardize the intensity values across the volume to the range [0, 1]. Next, we performed spatial interpolation to resize the axial dimension from 240 × 240 to 256 × 256 using trilinear interpolation. For the depth dimension, instead of processing all 155 slices, we used a center-focused slice selection strategy to capture the most relevant anatomical regions. We identified two central slices (slices 77 and 78 in the original volume) and then extracted 15 slices before the center (slices 62–76) and 15 slices after the center (slices 79–93), resulting in a total of 32 consecutive slices (15 + 2 + 15 = 32). This approach preserves critical tumor information typically located in the midbrain region while reducing computational complexity. The selected 32 slices are stacked along the depth dimension to form a unified 3D array with shape (32, 256, 256), which is then saved in NumPy (.npy) format. The BraTS caption text is encoded using the BioBERT model as described in the Multi-Modal Input Encoding section.

### Multi-Modal input encoding

At this stage, there are three different input modalities using different encoders that are appropriate for medical needs. First is the keyword extraction and embedding process. In this process, we use a KeyBERT model that has been fine-tuned specifically for medical use so that the model can extract clinically relevant keywords from radiology reports [12,13]. The extraction follows a four-level hierarchical structure that captures various aspects of lesion descriptions: (1) type of abnormality identifies pathological conditions such as glioblastoma, meningioma, or metastasis, (2) lesion characteristics describing appearance and intensity features including hyperintensity, heterogeneity, and contrast enhancement patterns, (3) anatomical location determining the affected brain region such as the frontal lobe, temporal lobe, or parietal cortex, and (4) lateralization indicating which hemisphere is affected: left, right, or bilateral. For each report, KeyBERT extracts the 5 most relevant keywords in these four categories based on semantic similarity. These multi-level keywords are then combined to produce a unified representation in the form [B, 1, 768] that captures semantic relationships.

Second, at this stage, researchers encoded 3D MRI images using the FLAIR modality. The processed 3D FLAIR MRI (32 × 256 × 256) was processed through the M3D Clip encoder in the previous data preprocessing stage [14]. This M3D Clip encoder uses a 3D convolution layer with spatial pooling to extract volumetric features, producing a global image representation with the form [B, 768]. This feature vector represents high-level anatomical and pathological patterns present in brain MRI, including tumor characteristics and surrounding tissue context.

Third, at this stage, the researchers performed ground-truth text encoding. The previously mentioned TextBraTS caption ground truth was tokenized and encoded using the BioBERT model [15]. We extracted the [CLS] token embedding to obtain a fixed-length representation with the form [B, 768] that captures the semantic content of the ground truth data description. Next, the three modalities keyword embedding [B, 1, 768], image embedding [B, 768], and text embedding [B, 768] use the same embedding space with a dimension of 768. By equalizing this dimension, the three types of data can be combined and processed together in the next cross-attention layer.

### Keyword-Guided with cross-attention

**Fig. 2:**
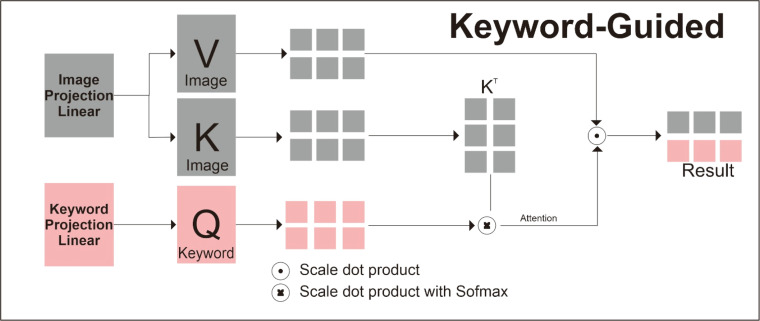
Keyword guided architecture model.

The cross-attention mechanism serves as the core fusion module that integrates visual features from MRI images with semantic information from clinical keywords As illustrated in Fig. 2.

In each keyword guide layer, the keyword embeddings serve as queries (Q) with shape [B, 1, 768], while the image features provide both keys (K) and values (V) with shape [B, 768]. This configuration allows the model to search through the entire image representation guided by clinical semantic information. The attention mechanism computes similarity scores between keyword queries and image keys, then uses these scores to aggregate relevant visual features from the values.

The attention operation follows the scaled dot-product formulation from [16]:

$$Attention\left(Q,K,V\right)=softmax\left(\frac{QK^{T}}{\sqrt{d_{k}}}\right)V$$

where $d_{k}$= 768 represents the dimension of the key vectors. The scaling factor $\sqrt{d_{k}}$ prevents the dot products from growing too large in high-dimensional spaces, which could lead to extremely small gradients during backpropagation. The softmax function normalizes the attention weights to sum to one, creating a probability distribution over the image features.

### Transformer decoder

The transformer decoder implements the standard architecture proposed by [16] through PyTorch library with syntax nn.TransformerDecoderLayer. The decoder consists of four stacked identical layers, where each layer contains three main sub-layers with specific computational roles.The first sub-layer performs masked multi-head self-attention over the target sequence. This mechanism computes attention between all positions in the output sequence, with masking applied to prevent positions from attending to subsequent positions. The multi-head attention with eight heads allows the model to jointly attend to information from different representation subspaces at different positions. Each head operates on dimension 96 (768/8), computing independent attention patterns that are concatenated and linearly projected back to dimension 768.

The second sub-layer implements multi-head cross-attention between the decoder and the encoder output. Here, the decoder queries attend to the enhanced multi-modal representations from the keyword-guided cross-attention module. This encoder-decoder attention mechanism enables the decoder to focus on relevant parts of the input conditioning while generating each token. The attention weights dynamically determine which aspects of the visual and semantic features are most relevant for predicting the next word.

The third sub-layer is a position-wise fully connected feed-forward network, which consists of two linear transformations with ReLU activation between them. The network applies the transformation $FFN\left(x\right)\;=\;max\left(0,\;xW_{1}+b_{1}\right)W_{2}+b_{2}$, where the inner layer expands from 768 to 2048 dimensions and the outer layer projects back to 768 dimensions. This feed-forward network is applied identically to each position separately.

### Training and parameter

The model is trained end-to-end to minimize cross-entropy loss between predicted and ground-truth caption tokens, with padding tokens ignored during computation. The loss function is formulated as:

$$L_{CE}=-T\frac{1}{t}=\sum_{t-1}^{T}\log P\left(yt|y<t,x\right)$$

where xrepresents the multi-modal input and $y_{t}$is the ground-truth token at position t.

We employ the AdamW optimizer with learning rate $5\;\times 10^{-5}$and weight decay 0.01 [17]. Training runs for 15 epochs with batch size 1, limited by the memory requirements of processing 3D FLAIR volumes (32 × 256 × 256) alongside large pre-trained models. The BraTS2020-TextBraTS dataset (369 patients) is split 80:20 for training and testing with patient-level stratification, yielding approximately 295 training and 74 test patients. Random seed 42 ensures reproducibility.

During training, we implement teacher forcing where target tokens are shifted by one position [18]. Token embeddings are scaled by $\sqrt{768}\;$and combined with sinusoidal positional encodings (max length 512, dropout 0.1). Maximum sequence length is set to 256 tokens. Causal masking through square subsequent mask ensures autoregressive generation, preventing the decoder from attending to future tokens.

Gradient clipping with maximum norm 1.0 stabilizes training through the deep architecture [19]. Model checkpoints are saved every 5 epochs, with the final model saved after epoch 15. A critical design choice is freezing the M3D-CLIP encoder weights, keeping it in evaluation mode throughout training [20]. Only the keyword-guided attention module (six cross-attention layers with 8 heads each), transformer decoder (four layers with feedforward dimension 2048), and output projection layer are optimized. The model is trained on an NVIDIA RTX 3050 GPU with 8GB VRAM and 32GB system RAM.

### Output generation and evaluation

During inference, the model generates radiological captions one word at a time sequentially. The process begins with a start token and continues by predicting the next word based on the words that have been generated previously. This process stops when the model generates an end token or reaches a maximum length of 256 tokens.The generated captions are evaluated using two metrics. The first metric is BLEU, which measures the precision of n-grams from the unigram level (BLEU-1) to the four-gram level (BLEU-4). BLEU-1 measures the accuracy of individual words, while BLEU-2 to BLEU-4 assess the match of longer word sequences. A higher BLEU score indicates better alignment with the reference. For medical reports, BLEU-1 above 0.4 and BLEU-2 above 0.3 are considered satisfactory given the variation in medical terminology expressions [21].

The second metric is ROUGE-L, which evaluates the longest common subsequence between the generated caption and the reference. ROUGE-L is more tolerant of word choice variations and focuses on the overall structural similarity of sentences [22]. Both metrics have a range of 0 to 1, with scores above 0.5 indicating excellent quality [23]. The evaluation was conducted by comparing each caption to the ground-truth radiological descriptions from TextBraTS, then averaging the scores across all test samples as a performance benchmark.

## Method validation

### Training performance

**Fig. 3:**
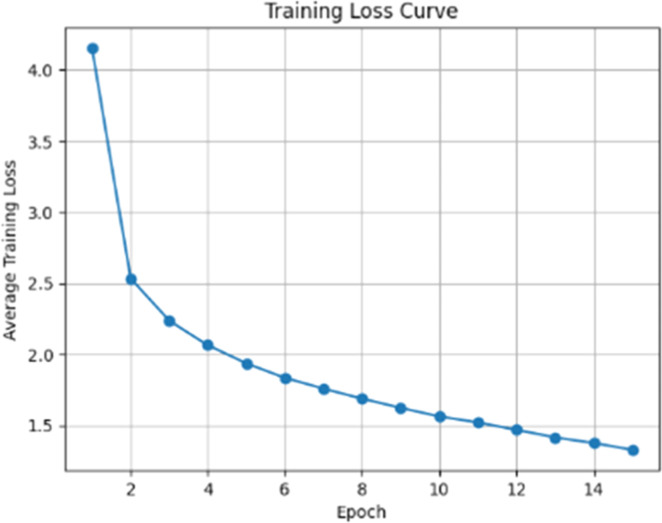
Visualization of training loss curve.

The model was trained for 15 epochs on the BraTS2020-TextBraTS dataset with a total of 295 training samples. As shown in Fig. 3 (Training Loss Curve), the loss curve shows consistent convergence during the training process, with the average loss gradually decreasing from 4.15 in the first epoch to 1.32 in the final epoch. A significant decrease in loss occurred in the early epochs, where the loss dropped from 4.1556 (epoch 1) to 2.0646 (epoch 4), indicating rapid learning by the model in understanding the basic patterns between visual features and text captions. After epoch 4, the loss continued to decrease at a slower gradient, reaching a value below 2.0 in epoch 5 (1.9365) and stabilizing around 1.5–1.8 for epochs 6–11. The final epoch shows further convergence with the loss reaching 1.3290, indicating that the model has learned to effectively represent the multimodal relationship between MRI images, clinical keywords, and radiological captions. All training was performed on an NVIDIA RTX 3050 GPU device, with an average processing speed of 3.5–3.8 iterations per second.

### Quantitative evaluation

A quantitative evaluation was conducted on 20 test samples using the BLEU and ROUGE-L metrics, with the summary results shown in Table 1. The model achieved a BLEU-1 score of 0.5359, which falls into the excellent performance category (>0.5) and far exceeds the satisfactory threshold for medical report generation (>0.4). This score indicates that the model has excellent word-level accuracy in generating captions that match the ground-truth reference. For higher n-grams, the model achieved a BLEU-2 score of 0.3969, a BLEU-3 score of 0.3009, and a BLEU-4 score of 0.2328. The BLEU-2 score almost reaches the good performance threshold (0.4), indicating that the model is capable of generating clinically accurate and relevant two-word sequences. The decrease in scores for BLEU-3 and BLEU-4 is common in text generation tasks, as these metrics are more sensitive to the exact matching of long sequences, and in the medical domain there is natural variation in how the same concepts are expressed. For the ROUGE metric, the model achieved a ROUGE-1 score of 0.6466, a ROUGE-2 score of 0.3634, and a ROUGE-L score of 0.5051. The ROUGE-L score of 0.5051 indicates strong performance (>0.5) in maintaining sentence structure and information flow consistent with the reference caption. This indicates that although there may be variations in specific word choices, the model is able to capture and reproduce the longest common subsequence that represents the correct organization of clinical information. The high ROUGE-1 (0.6466) further confirms that the model has excellent recall in identifying and including relevant keywords from the medical vocabulary.

**Table 1:** Metric evaluation.

Metric	BLEU-1	BLEU-2	BLEU-3	BLEU-4	ROUGE-1	ROUGE-2	ROUGE-L
Score	0.5359	0.3969	0.3009	0.2328	0.6466	0.3634	0.5051

### Qualitative analysis

Table 2 compares the ground truth and generated captions for Sample #1. The text marked in green indicates medical terminology and clinical information that was accurately identified by the model. Successfully identified elements include anatomical locations, namely the right frontal and parietal lobes; lesion signal characteristics, namely high and low signals and speckled high signals; edema distribution, namely the right parietal lobe and tissue swelling; necrosis findings, namely the right parietal and occipital lobes with low-signal intensity; and structural complications, namely ventricular compression. Although there are slight differences in specific phrases such as “mixed pattern” and “mixture of heterogeneous”.

**Table 2:** Comparisson between ground truth and generated captioning.

Name	Ground truth	Generated Captioning
Sample #1	The lesion area is in the right frontal and parietal lobes with a mixed pattern of high and low signals with speckled high signal regions. Edema is mainly observed in the right parietal lobe, partially extending to the frontal lobe, presenting as high signal, indicating significant tissue swelling around the lesion. Necrosis is within the lesions of the right parietal and frontal lobes, appearing as mixed, with alternating high and low signal regions. Ventricular compression is seen in the lateral ventricles with significant compressive effects on the brain tissue and ventricles.	the lesion area is in the right frontal and parietal lobes with a mixture of heterogeneous high and low signals, with speckled high signal areas. edema is significant, mainly observed in the right frontal and parietal lobes, with a large extent of swelling of the surrounding tissues. necrosis is observed in the right parietal and occipital lobes, characterized by low - signal intensity and mixed signals, concentrated in the right frontal and scattered low - signal intensity, displaying mixed signals. ventricular compression is observed, with the right ventricle

Overall, the evaluation results show that the proposed keyword-guided cross-attention architecture is effective in integrating visual information from 3D MRI with semantic guidance from hierarchical clinical keywords. The model demonstrates strong performance on lexical overlap metrics (BLEU-1 and ROUGE-L). The quality of the generated captions consistently captures key clinical information such as anatomical location, lesion characteristics, and edema distribution, which are important elements in brain tumor radiology reports.

## Limitations

This study has several methodological limitations. The model relies solely on the FLAIR modality, excluding T1ce and T2 sequences that reveal contrast-enhancing tumor core and peritumoral edema boundaries, limiting the clinical completeness of generated captions.

## Ethics statements

This study utilized publicly available datasets that are freely accessible to the research community. The 3D FLAIR MRI volumes were obtained from the Brain Tumor Segmentation (BraTS) 2020 Challenge dataset [24], and the corresponding radiological descriptions were sourced from the TextBraTS dataset [11]. Both datasets are distributed under open-access licenses for research purposes.

## CRediT author statement

**Supriyanto Supriyanto**: Software, Investigation, Data curation, Writing - Original draft preparation, Visualization. **Muhammad Ibadurrahman Arrasyid Supriyanto**: Conceptualization, Methodology, Supervision. **Haviluddin Haviluddin**: Methodology, Resources, Supervision. **Chalsi Mala Sari**: Supervision, Writing - Review & Editing. **Hajar Mar'atussholikah**: Investigation, Resources. **Rayner Alfred**: Conceptualization, Supervision. All authors have read and agreed to the published version of the manuscript.

## Supplementary material *and/or* additional information [OPTIONAL]

No.

## Declaration of competing interest

None.

## Data Availability

Data will be made available on request.
